# Sex Differences in the Prevalence of and Risk Factors for Abnormal Glucose Regulation in Adults Aged 50 Years or Older With Normal Fasting Plasma Glucose Levels

**DOI:** 10.3389/fendo.2020.531796

**Published:** 2021-02-19

**Authors:** Xinxin Zhang, Jie Liu, Shuang Shao, Yuan Yang, Dongwang Qi, Conglin Wang, Qiuxing Lin, Yue Liu, Jun Tu, Jinghua Wang, Xianjia Ning, Jingqiu Cui

**Affiliations:** ^1^ Department of Endocrinology and Metabolism, Tianjin Medical University General Hospital, Tianjin, China; ^2^ Department of Neurology, Tianjin Medical University General Hospital, Tianjin, China; ^3^ Laboratory of Epidemiology, Tianjin Neurological Institute, Tianjin, China; ^4^ Tianjin Neurological Institute, Key Laboratory of Post-Neuroinjury Neuro-repair and Regeneration in Central Nervous System, Ministry of Education and Tianjin City, Tianjin, China; ^5^ Department of Endocrinology and Metabolism, The Second Hospital of Tianjin Medical University, Tianjin, China; ^6^ Department of Geriatrics, Tianjin Medical University General Hospital, Tianjin, China

**Keywords:** sex differences, abnormal glucose regulation, prevalence, risk factors, epidemiology

## Abstract

**Aims:**

Abnormal glucose regulation, which can present as diabetes and prediabetes, has become one of the most common chronic conditions. However, sex differences in the prevalence of and factors associated with abnormal glucose regulation remain unclear. Thus, we aimed to explore sex differences in the prevalence of and factors associated with abnormal glucose regulation in low-income adults in China aged ≥50 years with normal fasting plasma glucose levels.

**Materials and Methods:**

A total of 2,175 individuals aged ≥50 years with normal fasting plasma glucose levels were recruited into this study. After an overnight fast of at least 10 h, individuals underwent an oral glucose tolerance test. Fasting and 2-h plasma glucose levels were measured to determine the state of glucose regulation.

**Results:**

Women were more likely than men to have isolated-impaired glucose tolerance (i-IGT) overall (24.7% vs 20.8%; P= 0.034), among individuals aged <65 years (21.7% vs 15.9%; P= 0.012). Among men, independent risk factors for i-IGT were an age of ≥65 years, hypertension, and high serum uric acid (SUA) and triglyceride levels; independent risk factors for diabetes mellitus (DM) were an age of ≥75 years and alcohol consumption. Among women, independent risk factors for i-IGT were central obesity and high levels of high-sensitivity C-reactive protein and SUA; independent risk factors for DM were low education and an elevated white blood cell count.

**Conclusions:**

Our findings suggest that conventional cardiovascular disease risk factors (i.e., age, hypertension, and dyslipidemia) associated with high risk of developing DM in men, but poor life style (i.e., obesity) and low education attainment in women. It is necessary for delay or stopping the development of DM among low-income adults in China to implement the personalized scheme of prevention DM between men and women, especially highlight control the risk factors in young and middle aged women.

## Introduction

It is well established that outcomes of abnormal glucose regulation include prediabetes and diabetes. Over the past few decades, diabetes has become one of the most common noncommunicable chronic diseases (1, 2). The number of adults with diabetes worldwide increased to 422 million in 2014, almost four times that in 1980 (2). The prevalence of diabetes and prediabetes in China has reached 10.9% and 35.7% in 2013, respectively (3), and the prevalence of prediabetes is higher among rural residents than among urban residents (3). In addition to the vascular diseases resulting from diabetes (4), diabetes is also associated with the risk of death from nonvascular conditions, such as cancers, infectious diseases, and degenerative disorders (5). Furthermore, mortality associated with diabetes has been shown to be higher in rural areas than in urban areas, even though there is a greater number of patients in urban districts (6).

Numerous studies have reported the prevalence of and risk factors for diabetes in adults (1, 6). Some previous studies have shown that men are more likely than women to develop diabetes, although there is some discrepancy in the factors reported to be associated with diabetes, including age, body mass index (BMI), dyslipidemia, low educational attainment, and hypertension (7–12). In contrast, several epidemiological studies have demonstrated that the prevalence of diabetes was higher in women than in men and that risk factors for women developing diabetes were age, waist circumference, BMI, hypertension, low educational attainment, large family size, living environment, while risk factors for men were age, BMI, and hypertension (13, 14). Previous studies have shown that the prevalence of isolated impaired glucose tolerance (i-IGT) was estimated to be between 6.2% and 9.3% (15–17). The Chinese nationally representative survey conducted in 2010 demonstrated that the prevalence of i-IGT was 11.0% in men and 10.9% in women and that type 2 diabetes is mainly manifested by elevated blood glucose after meals (18). More than half of the number of individuals with diabetes has isolated elevated 2-h plasma glucose (2-h PG) levels (19), which rise sharply with age (20). However, few studies have reported sex differences in the prevalence of and factors associated with abnormal glucose regulation in people with normal fasting plasma glucose (FPG) levels, especially among low-income adults with low educational attainment.

The routine examination in China focuses on FPG but rarely measures 2-h PG, leading to missed diagnoses of diabetes. The rural population is large in China, and sanitation is lacking, thus people with diabetes in rural areas are more likely to develop diabetic complications. It is necessary to recognize risk factors for abnormal glucose regulation among men and women with normal FPG levels to perform the oral glucose tolerance test (OGTT) in time and to develop personalized diabetic prevention plans for those with different risk factors. Thus, this study aimed to explore the sex differences in the prevalence of and factors associated with abnormal glucose regulation in adults from a low-income population in northern China aged ≥50 years with normal FPG levels.

## Materials and Methods

### Participants

This population-based, cross-sectional study was conducted between April 2019 and May 2019. The participants were adults aged ≥50 years from 18 administrative villages in Yangjinzhuang, a township at Ji County in Tianjin, China. Generally, this population has relatively low income and educational levels, and 95% are farmers, with a 2014 per capita disposable income of <1,600 USD (21). The exclusion criteria for the study were as follows: (1) a definite diabetes history identified by a positive answer from participants to the question “Has a doctor told you before that you have diabetes?” and/or (2) an FPG level ≥6.1 mmol/L and <7 mmol/L, obtained from medical records or participant report.

This study was approved by the medical research ethics committee of Tianjin Medical University General Hospital; written informed consent was obtained from recruited individuals before study participation.

### Data Collection

Trained researchers administered a standard questionnaire to participants that included information on education, occupation, income, medical history, and lifestyle characteristics. The interviewers were at local clinics in the participants’ residential areas and were trained according to a uniform standard. Demographic information, such as name, sex, and date of birth was obtained from existing records.

Lifestyle characteristics included cigarette smoking, alcohol consumption, physical exercise, and sleep duration. Cigarette smoking was defined as smoking >1 cigarette/day for at least 1 year; participants were categorized as non-smokers, former smokers (defined as those who had stopped smoking for at least 6 months), and current smokers. Alcohol consumption was defined as drinking more than 500 g alcohol/week >1 year; participants were categorized as non-drinkers, former drinkers (defined by temperance for at least 6 months), and current drinkers. Physical exercise was defined as being involved in moderate or vigorous activity ≥30 min/day for at least 3 days per week. Sleep duration was based on self-report. Participants were categorized into four groups according to sleep duration: <5 h, 5–6 h, 7–9 h, and >9 h.

### Measurements

Physical examinations, including assessment of height, weight, waist circumference, and blood pressure (BP), were performed by trained physicians at the village clinics. Anthropometric measurements were conducted with participants wearing light clothing without a hat or shoes. Height and weight were measured while the participant was in a fully vertical position using a calibrated instrument, zeroed before each measurement. Waist circumference was measured with a non-stretching measuring tape placed on the participant’s horizontal plane at the midpoint between the top of the iliac crest and the bottom of the costal margin in the midaxillary line. BP, including systolic BP (SBP) and diastolic BP (DBP), was obtained using an electronic sphygmomanometer; participants were asked to take off their coats and roll up their sleeves to the shoulders after 5 min of quiet rest. Two measurements were made 5 min apart, and the second measurement was the reported value.

Before undergoing the oral glucose tolerance test (OGTT), participants were asked to maintain their daily physical activity and diet for at least 3 days. After participants had fasted overnight for at least 10 h, they had venous blood samples taken for measurement of FBP, total cholesterol (TC), triglycerides (TG), high-density lipoprotein cholesterol (HDL-C), low-density lipoprotein cholesterol (LDL-C), high-sensitivity C-reactive protein (hs-CRP), and serum uric acid (SUA) levels. Participants then drank a standard 75-g glucose solution, and venous blood samples were drawn 120 min later for the measurement of 2-h PG. All blood samples were immediately delivered to Guangzhou KingMed Diagnostics Group Co., Ltd., an Independent Clinical Laboratories industry in China, for all measurements.

Carotid ultrasonography was performed using a Mindary M7 portable ultrasound system. A professional radiologist used a scanner to scan and store images of both common carotid arteries, both carotid sinuses, and both proximal internal carotid arteries. Later, carotid intima-media thickness (CIMT) was measured by two experienced imaging physicians based on blood vessel images.

### Definitions

BMI was calculated as the participant’s weight (in kg) divided by the square of the participant’s height (m^2^). According to the BMI, participants were categorized as underweight (BMI ≤ 18.5 kg/m^2^), normal weight (18.5 kg/m^2^ <BMI< 24.0 kg/m^2^), overweight (24.0 kg/m^2^ ≤ BMI< 28.0 kg/m^2^), or obese (BMI ≥28 kg/m^2^) (22). Central obesity was defined as a waist circumference >90 cm for men and >85 cm for women (23). Hypertension was defined as an SBP ≥140 mmHg, DBP ≥90 mmHg, or for participants taking antihypertension medications. Diabetes was defined as an FPG level ≥7.0 mmol/L, a 2-h PG level ≥11.1 mmol/L, a previous history of diagnosed diabetes, or by the use of hypoglycemic drugs (24). Individuals with normal FPG levels were categorized as having normal glucose tolerance (NGT, 2-h PG level <7.8 mmol/L), isolated-impaired glucose tolerance (i-IGT, 2-h PG level 7.8-11.0 mmol/L), or diabetes mellitus (DM, 2-h PG level ≥11.1 mmol/L).

### Statistical Analyses

Continuous variables, including age, number of years of formal education, BMI, SBP, DBP, FPG level, 2-h PG level, white blood cell (WBC) count, hs-CRP level, SUA level, TC level, TG level, HDL-C level, LDL-C level, and CIMT, are presented as means with standard deviations and were compared between NGT and i-IGT/DM groups using Student’s t-tests. Categorical variables, such as age group, education group, income group, smoking status, alcohol consumption status, the number of days per week that a participant engaged in physical activity, and hypertension status, are presented as numbers with frequencies and were compared between NGT and i-IGT/DM groups using chi-squared tests. Binary logistic regression analyses were used to evaluate the associations between DM and i-IGT prevalence with those factors found to be statistically significant in the univariate analysis. The associations are presented as odds ratios (ORs) and 95% confidence intervals (CIs). P-values <0.05 in the two-tailed tests were considered statistically significant. SPSS for Windows (version 22.0; IBM Corp., Armonk, NY, USA) was used for analyses.

## Results

A total of 2,647 persons were recruited to the study, and 472 were excluded for having an elevated FPG level (FPG ≥6.1 mmol/L); thus, 2,175 participants were included in the final analyses.

### Demographic Characteristics

In this study, 53.9% (n = 1,172) were women, and 54.6% (n = 1,187) were individuals aged 50–65 years. The mean number of years of formal education was only 5.53 years overall, and 97.2% of participants had an annual income <6,000 yuan. Among participants, 74.6% had hypertension, and 64.6% were overweight or obese. The mean SBP and DBP were 149.03 mmHg and 84.66 mmHg, respectively, and the FPG and 2-h PG levels were 5.26 mmol/L and 6.75 mmol/L, respectively (**Table 1**).

**Table 1:** Demographic and clinical characteristics of all participants.

Category	Total	Men	Women	P
Participants, n, (%)	2,175(100)	1,003 (46.1)	1,172 (53.9)	<0.001
Age, means (SD), years	64.46 (7.68)	65.65 (7.59)	63.44 (7.61)	
Age groups, n (%)				<0.001
<65 years	1,187 (54.6)	497 (49.6)	690 (58.9)	
65 years∼	788 (36.2)	392 (31.9)	396 (33.8)	
≥75 years	200 (9.2)	114 (11.4)	86 (7.3)	
Education, means (SD), years	5.53 (3.55)	6.64 (2.90)	4.58 (3.72)	<0.001
Education, n (%),				<0.001
≤6 years	1,277 (58.7)	495 (49.4)	782 (66.7)	
> 6 years	898 (41.3)	508 (50.6)	390 (33.3)	
Income, n, (%)				0.569
< 2,000 yuan	1,444 (66.4)	677 (67.5)	767 (65.4)	
2,000∼6,000 yuan	670 (30.8)	300 (29.9)	370 (31.6)	
> 6,000 yuan	61 (2.8)	26 (2.6)	35 (3.4)	
Smoking status, n (%)				<0.001
Never smoking	1,350 (62.7)	242 (24.5)	1,108 (95.3)	
Ever smoking	259 (12.0)	248 (25.1)	11 (0.9)	
Current smoking	543 (25.2)	499 (50.5)	44 (3.8)	
Alcohol consumption, n, (%)				<0.001
Never drinking	1,597 (74.0)	454 (28.4)	1,143 (97.9)	
Ever drinking	131 (6.1)	124 (5.7)	7 (0.6)	
Current drinking	431 (20.0)	414 (41.7)	17 (1.5)	
sleep duration, n, (%)				<0.001
<5 h	408 (19.0)	111 (11.2)	297 (25.5)	
5∼ h	639 (29.7)	285 (28.8)	354 (30.4)	
7∼ h	972 (45.1)	518 (52.4)	454 (39.0)	
>9 h	134 (6.2)	74 (7.5)	60 (5.2)	
Physical exercise, n, (%)				0.388
No	844 (38.8)	399 (39.8)	445 (38.0)	
Yes	1331 (61.2)	604 (60.2)	727 (62.0)	
Hypertension, n, (%)				0.132
No	552 (25.4)	239 (23.9)	313 (26.8)	
Yes	1,617 (74.6)	760 (76.1)	857 (73.2)	
BMI, means (SD), Kg/m2	25.45 (3.63)	24.88 (3.34)	25.94 (3.80)	<0.001
BMI, n, (%)				<0.001
underweight	37(1.7)	15 (1.5)	22(1.9)	
Normal	768 (35.4)	396 (39.5)	335 (28.7)	
Over weight	932 (42.9)	428 (42.7)	504 (43.1)	
Obesity	472 (21.7)	164 (16.4)	308 (26.3)	
Central obesity, n, (%)				<0.001
No	1,245 (57.3)	658 (65.6)	587 (50.2)	
Yes	927 (42.7)	345 (34.4)	582 (49.8)	
SBP, means (SD), mmHg	149.03 (20.00)	150.38 (20.64)	147.88 (19.38)	0.004
DBP, means (SD), mmHg	84.66 (10.92)	87.04 (11.40)	82.64 (10.06)	<0.001
FPG, means (SD), mmol/L	5.26 (0.44)	5.27 (0.45)	5.26 (0.43)	0.678
2-h PG, means (SD), mmol/L	6.75 (2.00)	6.49 (2.16)	6.97 (1.83)	<0.001
WBC, means (SD), ×10^9^/L	6.05 (1.60)	6.20 (1.66)	5.93 (1.54)	<0.001
hs-CRP, means (SD), mg/L	2.20 (4.04)	2.20 (3.94)	2.20 (4.13)	0.991
SUA, means (SD), μmmol/L	298.08 (84.85)	330.01 (86.70)	270.75 (72.90)	<0.001
TC, means (SD), mmol/L	5.11 (0.88)	4.90 (0.81)	5.29 (0.89)	<0.001
TG, means (SD), mmol/L	1.47 (1.02)	1.33 (1.03)	1.60 (0.99)	<0.001
HDL-C, means (SD), mmol/L	1.43 (0.44)	1.42 (0.47)	1.43 (0.41)	0.661
LDL-C, means (SD), mmol/L	3.10 (0.86)	2.95 (0.79)	3.24 (0.88)	<0.001
CIMT, means (SD), µm	0.71 (0.14)	0.73 (0.14)	0.70 (0.13)	<0.001

SD, standard deviation; BMI, body mass index; SBP, systolic blood pressure; DBP, diastolic blood pressure; FPG, fasting plasma glucose; 2-h PG, two-hour plasma glucose; WBC, white blood cell; hs-CRP, high-sensitivity C-reactive protein; SUA, serum uric acid; TC, total cholesterol; TG, triglycerides; HDL-C, high density lipoprotein cholesterol; LDL-C, low density lipoprotein cholesterol; CIMT, carotid intima-media thickness.

### Sex Differences in the Prevalence of i-IGT and DM

Among all participants, women were more likely than men to have i-IGT (24.7% vs. 20.8%; P = 0.034), and this sex difference was also true among individuals aged <65 years (P<0.05). There were no significant sex differences in DM prevalence among all participants or by education or income subgroup (P>0.05; **Table 2**).

**Table 2:** Sex differences in the prevalence of abnormal glucose regulation by demographic characteristics.

Groups	i-IGT			DM		
	Men	Women	P	Men	Women	P
Participants, n (%)	209 (20.8)	289 (24.7)	0.034	32 (3.2)	33 (2.8)	0.609
Age groups, n (%)						
<65years	79 (15.9)	150 (21.7)	0.012	11 (2.2)	14 (2.0)	0.827
65∼years	93 (23.7)	108 (27.3)	0.253	14 (3.6)	15 (3.8)	0.872
≥75years	37 (32.5)	31 (36.0)	0.596	7 (6.1)	4 (4.7)	0.647
Education, n (%),						
≤6 years	126 (25.5)	206 (26.3)	0.724	16 (3.2)	28 (3.6)	0.740
>6 years	83 (16.3)	83 (21.3)	0.059	16 (3.1)	5 (1.3)	0.066
Income, n, (%)						
<2,000 yuan	150 (22.2)	204 (26.6)	0.050	21 (3.1)	22 (2.9)	0.794
2,000∼6,000 yuan	54 (18.0)	81 (21.9)	0.212	11 (3.7)	11 (3.0)	0.616
>6,000 yuan	5 (19.2)	4 (11.4)	0.395	0 (0.0)	0 (0.0)	—

### Risk Factors for Abnormal Glucose Regulation in Men and Women

**Table 3** shows that, among men, the prevalence of i-IGT increased with increasing age, BP, and BMI (P<0.05), and the prevalence of i-IGT was significantly higher among those whose formal education was ≤6 years and who had central obesity (P<0.001). Older men and those who consumed alcohol had a greater risk of DM than did those <65 years of age and those who had never consumed alcohol (P<0.05). Among women, the prevalence of i-IGT was greater in those with hypertension and obesity (P<0.001). Women who had less formal education and less income were at an increased risk of i-IGT (P= 0.035). Furthermore, women with ≤6 years of education had a higher risk of DM (P= 0.016), and the prevalence of DM was higher among women with hypertension and central obesity (P<0.05).

**Table 3:** Sex differences in the risk factors of abnormal glucose regulation in men and women.

Groups	Men		Women	
	I-IGT	DM	I-IGT	DM
Age groups, n (%)				
< 65 years	79 (16.3)*	11 (2.6)*	150 (22.2)*	14 (2.6)
65∼years	93 (24.6)	14 (4.7)	108 (28.3)	15 (5.2)
≥75 years	37 (34.6)	7 (9.1)	31 (37.8)	4 (7.3)
Education, n (%),				
≤6 years	126 (26.3)*	16 (4.3)	206 (27.3)*	28 (4.9)*
> 6 years	83 (16.9)	16 (3.8)	83 (21.6)	5 (1.6)
Income, n, (%)				
< 2,000 yuan	150 (22.9)	21 (4.0)	204 (27.4)*	22 (3.9)
2,000∼6,000 yuan	54 (18.7)	11 (4.5)	81 (22.6)	11 (3.8)
> 6,000 yuan	5 (19.2)	0 (0.0)	4 (11.4)	0 (0.0)
Smoking status, n (%)				
Never smoking	58 (24.4)	4 (2.2)	278 (25.8)	32 (3.9)
Ever smoking	58 (24.4)	10 (5.3)	2 (18.2)	0 (0.0)
Current smoking	90 (18.7)	18 (4.4)	6 (14.0)	1 (2.6)
Alcohol consumption, n, (%)				
Never drinking	95 (21.3)	7 (1.9)*	285 (25.7)	33 (3.8)
Ever drinking	32 (26.7)	4 (4.3)	1 (14.3)	0 (0.0)
Current drinking	80 (20.4)	21 (6.3)	2 (11.8)	0 (0.0)
sleep duration, n, (%)				
< 5 h	24 (22.6)	5 (5.7)	80 (28.1)	12 (5.5)
5∼ h	52 (18.8)	9 (3.9)	86 (25.1)	11 (4.1)
7∼ h	115 (22.8)	13 (3.2)	107 (24.0)	8 (2.3)
> 9 h	15 (21.7)	5 (8.5)	13 (22.0)	1 (2.1)
Physical exercise, n, (%)				
No	69 (17.7)*	10 (3.0)	104 (24.1)	13 (3.8)
Yes	140 (24.1)	22 (4.7)	185 (26.2)	20 (3.7)
Hypertension, n, (%)				
No	27 (11.5)*	4 (1.9)	56 (18.1)*	4 (1.6)*
Yes	181 (24.7)	27 (4.7)	233 (28.1)	29 (4.6)
BMI, n, (%)				
underweight	1(6.7)*	0 (0)	5(22.7)*	0 (0)
Normal	62 (16.2)	13 (3.7)	66 (20.2)	8 (2.8)
Over weight	101 (24.5)	16 (4.9)	124 (25.2)	11 (2.9)
Obesity	45 (28.0)	3 (2.5)	93 (31.6)	14 (6.5)
Central obesity, n, (%)				
No	116 (18.1)*	18 (3.3)	113 (19.6)*	10 (2.1)*
Yes	93 (28.1)	14 (5.6)	175 (31.3)	23 (5.7)

*indicated P < 0.05; NGT, normal glucose tolerance; i-IGT, isolated impaired glucose tolerance; DM, diabetes mellitus; BMI, body mass index.

As shown in **Table 4**, men with elevated WBC counts, hs-CRP levels, SUA levels, TC levels, TG levels, and increased CIMT had an increased risk of i-IGT (P<0.05). Additionally, men with high levels of SUA and increased CIMT had an increased risk of DM (P<0.05). Women with i-IGT had lower HDL-C levels, higher WBC counts, hs-CRP, SUA, TG, and CIMT than those with NGT did (P<0.05). The pattern of association described above for HDL-C and WBC was also consistent for women with DM (P<0.05).

**Table 4:** Sex differences of serology and CIMT with abnormal glucose regulation in men and women.

Groups	Men		Women	
	I-IGT	DM	I-IGT	DM
WBC, ×10^9^/L	6.55 (2.21)*	6.02 (1.50)	6.26 (1.63)*	6.58 (1.62)*
hs-CRP, mg/L	2.95 (5.27)*	2.54 (4.32)	3.10 (6.03)*	2.62 (2.98)
SUA, μmmol/L	363.89 (97.96)*	351.84 (103.39)*	288.54 (80.51)*	274.88 (62.64)
TC, mmol/L	5.01 (0.94)*	4.97 (0.90)	5.35 (0.92)	5.39 (0.76)
TG, mmol/L	1.60 (1.70)*	1.30 (0.62)	1.75 (0.98)*	1.85 (0.90)
HDL-C, mmol/L	1.37 (0.46)	1.50 (0.55)	1.37 (0.38)*	1.30 (0.29)*
LDL-C, mmol/L	3.00 (0.84)	2.95 (0.81)	3.31 (0.92)	3.37 (0.73)
CIMT, µm	0.75 (0.15)*	0.77 (0.15)*	0.71 (0.13)*	0.72 (0.13)

*indicated P < 0.05; NGT, normal glucose tolerance; i-IGT, isolated impaired glucose tolerance; DM, diabetes mellitus; WBC, white blood cell; hs-CRP, high-sensitivity C-reactive protein; SUA, serum uric acid; TC, total cholesterol; TG, triglycerides; HDL-C, high density lipoprotein cholesterol; LDL-C, low density lipoprotein cholesterol; CIMT, carotid intima-media thickness.

### Risk Factors Identified in the Multivariate Analysis for Abnormal Glucose Regulation in Men

**Figure 1:**
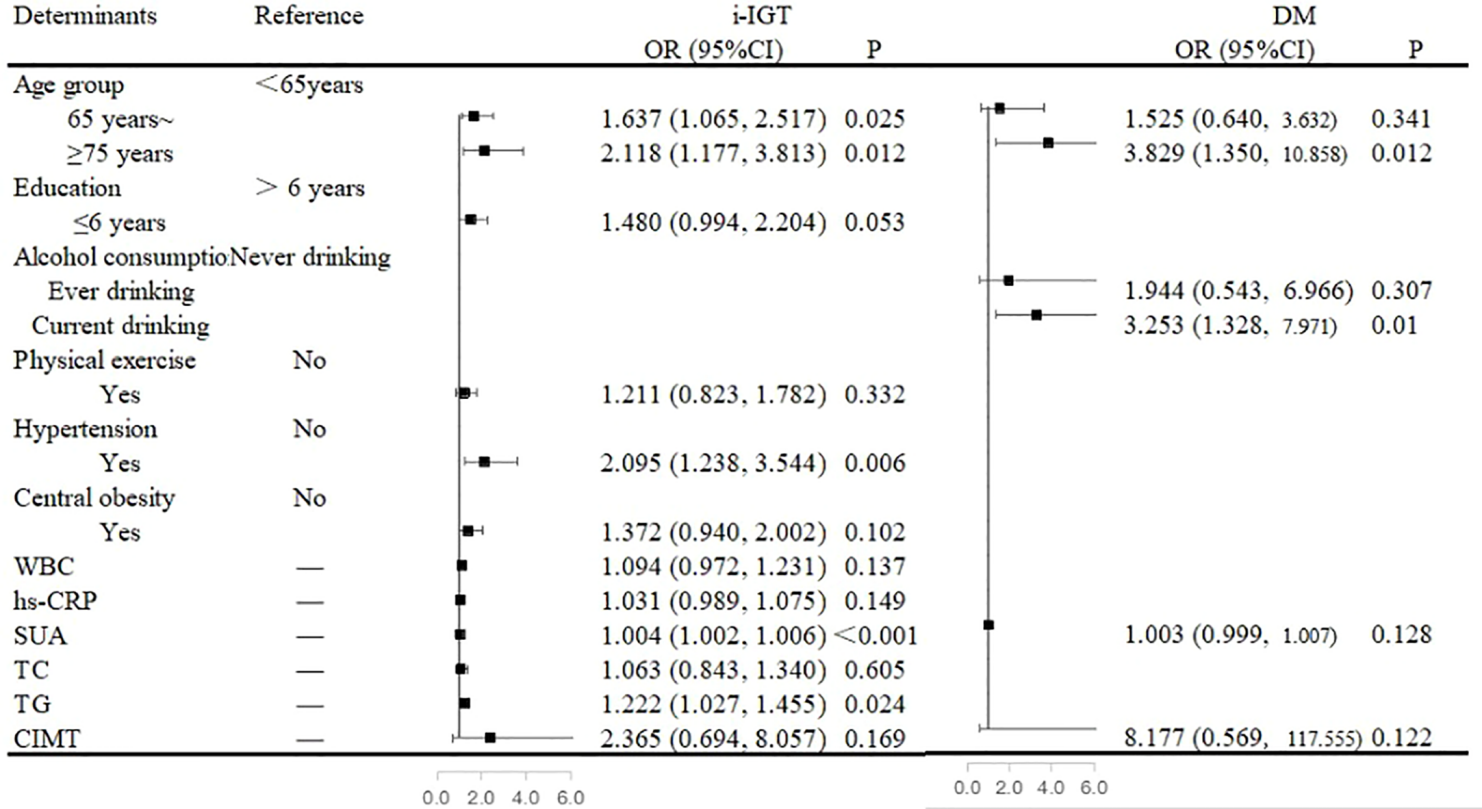
Associated factors of abnormal glucose regulation in the multivariate analyses in men. **Figure 1** showed that an age of ≥65 years, the presence of hypertension, and high serum uric acid (SUA) and triglycerides (TG) levels were independent risk factors for isolated-impaired glucose tolerance (i-IGT) among men. An age of ≥75 years and alcohol consumption were independent risk factors for diabetes mellitus (DM) among men.

In the multivariate analysis, an age of ≥65 years, the presence of hypertension, and high SUA and TG levels were independent risk factors for i-IGT among men. When compared with that among men <65 years of age, the prevalence of i-IGT was 63.7% higher among men who were in the 65–74-year age group (OR, 1.637; 95% CI, 1.065–2.517; P= 0.025) and 111.8% higher among men in the ≥75-year age group (OR, 2.118; 95% CI, 1.177–3.813; P= 0.012). Men with hypertension had a 109.5% increase in the prevalence of i-IGT compared with men with normal BP (OR, 2.095; 95% CI, 1.238–3.544; P= 0.006). Furthermore, the prevalence of i-IGT among men increased by 4% with each 1-µmol/L increase in SUA level (OR, 1.004; 95% CI, 1.002-1.006; P<0.001) and by 22.2% with each 1-mmol/L increase in TG level (OR, 1.222; 95% CI, 1.027–1.455; P= 0.024; **Figure 1**).

An age of ≥75 years and alcohol consumption were independent risk factors for DM among men. When compared with that among men <65 years of age, the prevalence of DM was almost three-fold higher among men aged ≥75 years (OR, 3.829; 95% CI, 1.350–10.858; P= 0.012). Moreover, alcohol consumption was associated with a 225.3% increased risk of DM among men (OR, 3.253; 95% CI, 1.328-7.971; P= 0.010; **Figure 1**).

### Risk Factors Identified in the Multivariate Analysis for Abnormal Glucose Regulation in Women

**Figure 2:**
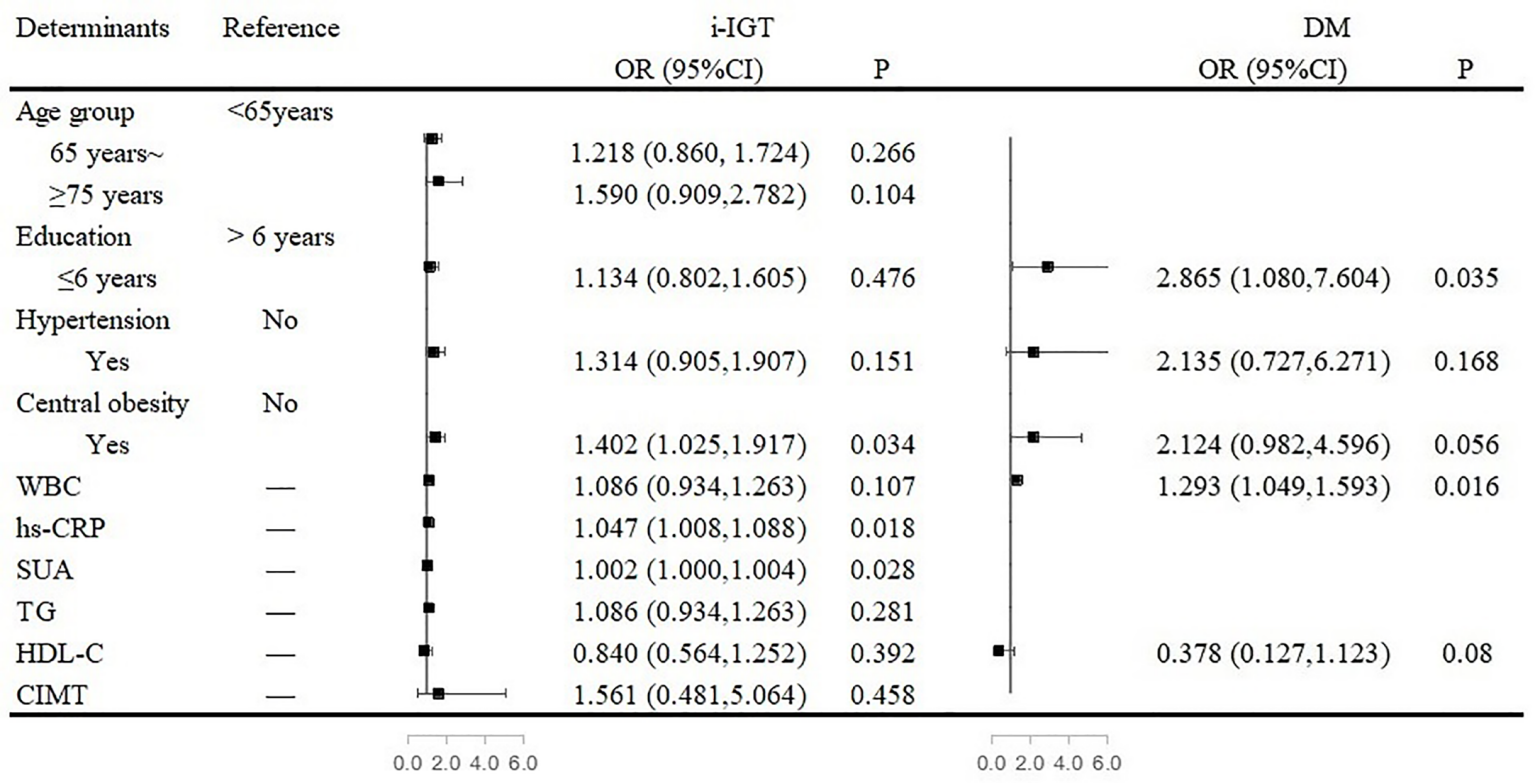
Associated factors of abnormal glucose regulation in the multivariate analyses in women. **Figure 2** showed that central obesity and high levels of high-sensitivity C-reactive protein (hs-CRP) and serum uric acid (SUA) were independent risk factors for isolated-impaired glucose tolerance (i-IGT). Low education and elevated white blood cell (WBC) count were independent risk factors for diabetes mellitus (DM) among women.

Among women, central obesity and high levels of hs-CRP and SUA were independent risk factors for i-IGT. The prevalence of i-IGT was 40.2% higher for women who had central obesity than for women who did not (OR, 1.402; 95% CI, 1.025–1.917; P= 0.034). Each 1-µmol/L increase in SUA level increased the prevalence of i-IGT by 2% among women (OR, 1.002; 95% CI, 1.000-1.004; P= 0.028). Similarly, women with a high level of hs-CRP were at greater risk of having i-IGT (OR, 1.047; 95% CI, 1.008–1.088; P= 0.018; **Figure 2**).

Low education and elevated WBC count were independent risk factors for DM among women. Women who received a formal education of <6 years had a 186.5% higher risk of DM than did women with longer formal educations (OR, 2.865; 95% CI, 1.080–7.604; P<0.001). Furthermore, for every 1 × 10^9^/L increase in WBC count, the risk of DM increased by 29.3% (OR, 1.293; 95% CI, 1.049–1.593; P= 0.016; **Figure 2**).

## Discussion

In this cross-sectional study, we explored sex differences in the prevalence of and risk factors associated with abnormal glucose regulation in low-income adults aged 50 years or older in northern China with normal FPG levels. Women were more likely than men overall to develop i-IGT, and this was also true for individuals aged <65 years; there was no significant sex-based difference in DM prevalence. Among men, an age of ≥65 years, hypertension, and high SUA and TG levels were independent risk factors for i-IGT; an age of ≥75 years and alcohol consumption were independent risk factors for DM. Among women, central obesity and high levels of hs-CRP and SUA were independent risk factors for i-IGT; low educational attainment and an elevated WBC count were independent risk factors for DM.

The prevalence of diabetes in China has increased sharply in the past few decades (25), from 2.5% in 1994 (26) to 10.9% in 2013 (3). Some studies reporting sex differences in the prevalence of diabetes reported that women were more susceptible to diabetes than were men (14). A meta-analysis associated with inland residents in China showed that the prevalence of diabetes was higher among women than among men (11.6% vs 9.3%) (25). However, other studies suggested that men were more likely to have diabetes (7–9, 27). Similarly, a cross-sectional study of 46,239 individuals in China showed that male sex was an independent risk factor for diabetes (OR, 1.26; 95% CI, 1.12–1.43) (18). The National Health and Nutrition Examination Surveys in America demonstrated that the prevalence of prediabetes was higher in women than in men among adults aged ≥50 years (28). In the present study, we observed that women were more likely overall to have i-IGT and that this was also true among individuals aged <65 years. In contrast, we did not observe a significant sex-based difference in DM prevalence. Different study designs and populations may explain these discrepant conclusions. It is well-known that women usually live longer than men; therefore, it may be reasonable to devote more attention to understanding women with prediabetes and diabetes in order to promote their health, especially those with a low income and aged <65 years.

Many studies have explored the association between advanced age and diabetes (25, 29). A 12-year follow-up study that enrolled individuals aged 40–69 years reported that age was an independent risk factor for progression to diabetes (HR, 1.02; 95% CI, 1.02–1.03) (30). Similarly, the China National Diabetes and Metabolic Disorders Study, conducted from June 2007 to May 2008, demonstrated that older age was significantly associated with an increased risk of diabetes (18). In accordance with the results of previous studies, we concluded that advanced age was an independent risk factor for i-IGT (≥65 years) and diabetes (≥75 years), but among men alone in this low-income population. The decrease in insulin secretion, especially in people with impaired glucose tolerance, may partly explain the effect of advanced age on diabetes and prediabetes (31).

Previous reports on the association between education and diabetes are inconsistent. The China Kadoorie Biobank study, which included 0.5 million adults recruited from 10 diverse areas in China, concluded that, compared with having no formal education, having a college education or higher was associated with a 21% increase in the prevalence of diabetes (OR, 1.21; 95% CI, 1.09–1.35) and a 27% increase in the incidence of diabetes (HR, 1.27; 95% CI, 1.07–1.51) among men, whereas having a college education or higher was associated with a 31% decrease in the prevalence of diabetes (OR, 0.69; 95% CI, 0.63–0.76) and a 20% decrease in the incidence of diabetes (HR, 0.80; 95% CI, 0.67–0.95) among women (32). However, there are also some studies hold the opposite view (27, 33). A prospective study in low-, middle-, and high-income countries, including China, revealed that a low education level was an independent risk factor for diabetes (OR, 1.10; 95% CI, 1.02-1.19) (33). In the current study, low education was an independent risk factor for diabetes among women alone. Similar to socioeconomic status, level of education can affect health through complex mechanisms, such as health care, environmental exposure, and health behavior (34).

The association between alcohol consumption and the risk of developing DM remains controversial. People currently consuming 8–14 drinks/week have been reported to have a lower risk of diabetes than did those consuming ≤1 drink/week for both men (HR, 0.84; 95% CI, 0.70–1.00) and women (HR, 0.71; 95% CI, 0.56–0.91) (35). However, reductions in the risk of developing diabetes among moderate drinkers appears to apply to women and non-Asian populations alone (36). Studies in Asia, including China and Japan, suggest that regular drinkers and heavy drinkers have an increased risk of developing diabetes than do social drinkers (37, 38). Even for moderate drinkers, a higher alcohol intake increased the risk of developing diabetes (39). In accordance with results of the studies mentioned above, current drinking was found to be an independent risk factor for i-IGT among men in this cross-sectional study.

Obesity and central obesity are accepted risk factors for diabetes (7–9, 40). Central obesity has been shown to significantly increase the risk of prediabetes (OR, 1.22; 95% CI, 1.06–1.40) and diabetes (OR, 1.39; 95% CI, 1.18–1.63) (18). In this study, women with central obesity were at a higher risk of having i-IGT. In humans with obesity, reduced G protein pathway suppressor 2 (GPS2) expression in macrophages causes elevated systemic and adipose tissue inflammation, insulin resistance, and diabetes (41, 42). These changes may contribute to the greater risk of IGT in those who are obese, but the cause of the sex disparity remains unclear.

The positive association between hypertension and diabetes has been established in previous studies (18, 29). A 10-year, community-based, prospective study in Korea demonstrated that individuals with prehypertension, stage 1 hypertension, and stage 2 hypertension had a 23%, 26%, and 60% increased risk of diabetes, respectively, compared with individuals with normal blood pressure (<120/80 mmHg) (43). In addition, usual blood pressure (95 mmHg <SBP ≤195 mmHg; 65 mmHg <DBP ≤115 mmHg) also increased the risk of new-onset diabetes; each 20-mmHg elevation in SBP and 10-mmHg elevation in DBP increased the risk of new-onset diabetes by 58% and 52%, respectively, when compared with BP within the normal range (44). Among men in this low-income population, hypertension was an independent risk factor for i-IGT. The links between obesity, diabetes, and BP may be attributed to adipokine dysregulation in perivascular adipose tissue (45).

Previous studies have also reported a positive association between TG level and diabetes (46–48).In the present study, we found that TG level was an independent risk factor for i-IGT among men, resulting in a 23.4% increase in the prevalence of i-IGT with each 1-mmol/L increase in TG level. The association between TG and diabetes may be partly mediated by insulin resistance and increased insulin secretion (49, 50). As a result, lipid management is essential, especially for those with chronic diseases.

Diabetes is an inflammatory disease (51). In a cross-sectional study of a Chinese population, there was a U-shaped association between WBC count and the incidence of diabetes (52). However, other studies have indicated that WBC count increased the risk of incident diabetes, even when it was within the normal range (53). A substudy of working-class individuals in Hong Kong indicated that WBC count was an independent predictor for hyperglycemia and increased insulin resistance among Chinese men (54). In the present study, WBC count was an independent risk factor for diabetes in women. Decreased insulin sensitivity and incidence of diabetes mediated by elevated WBC count may partly explain this association (55).

hs-CRP is a major acute-phase protein marking systematic inflammation in chronic disease. Some studies have demonstrated a positive association between hs-CRP level and diabetes (56–58). Similarly, we concluded that women with high levels of hs-CRP were at an increased risk of i-IGT in this low-income population. hs-CRP level has been shown to predict changes in insulin sensitivity and future insulin resistance even in non-diabetic adults; thus, hs-CRP level may indicate the imminent development of type 2 diabetes (59, 60).

Higher levels of uric acid were associated with an increased risk of diabetes, both in quantitative and qualitative analyses (61–64). Consistent with the results of these studies, uric acid level was an independent risk factor for i-IGT in both sexes in this low-income population. This association may be explained by higher levels of uric acid inducing oxidative stress, which favors the development of diabetes (65, 66).

There are several limitations of this cross-sectional study. First, we did not measure insulin levels, so we could not evaluate insulin resistance or function of islet beta-cells. Second, we explored the prevalence of abnormal glucose regulation and associated risk factors using people living in Tianjin rural areas; therefore, our findings cannot be generalized to other populations. Third, because this was a cross-sectional study, we could not identify causal associations. Fourth, nutritional factors, as important risk factors for diabetes, were not measured in this low-income population. Finally, OGTT was not performed in people with impaired fasting glucose and known diabetes. The conclusions of the study may be weak without the data of impaired fasting glucose and known diabetes. Therefore, we plan to perform OGTT under careful management among all participants in future research.

## Conclusion

In this population-based, cross-sectional study, women were more likely than men to develop i-IGT overall, and this was also true for individuals aged <65 years. Our findings suggest that conventional cardiovascular disease risk factors (i.e., age, hypertension, and dyslipidemia) are associated with a high risk of developing DM in men, while poor life style (i.e., obesity) and low education attainment are risk factors in women. It is necessary to implement the personalized scheme between men and women for delay or stopping the development of DM and to perform OGTT for early diagnosis of diabetes in men and women with multiple risk factors, especially women with a low income and aged less than 65 years.

## Data Availability Statement

The datasets generated for this study are available on request to the corresponding authors.

## Ethics Statement

This study was approved by the medical research ethics committee of Tianjin Medical University General Hospital. The patients/participants provided their written informed consent to participate in this study.

## Author Contributions

JC, JW, and XN were involved in conception, design, and data collection. XZ and JL were involved in manuscript drafting for this article. XZ, JL, SS, YY, DQ, CW, QL, YL, JT, JW, XN, and JC were involved in data collection and case diagnosis for this article. JW and XN were involved in data analysis, and data interpretation. JC, JW, and XN were involved critical review for this article. All authors contributed to the article and approved the submitted version.

## Conflict of Interest

The authors declare that the research was conducted in the absence of any commercial or financial relationships that could be construed as a potential conflict of interest.

The reviewer XY declared a shared affiliation with the authors, to the handling editor, at time of review.
